# Human Calmodulin-Like Protein CALML3: A Novel Marker for Normal Oral Squamous Mucosa That Is Downregulated in Malignant Transformation

**DOI:** 10.1155/2013/592843

**Published:** 2013-07-08

**Authors:** Michael D. Brooks, Richard D. Bennett, Amy L. Weaver, Thomas J. Sebo, Steven E. Eckert, Emanuel E. Strehler, Alan B. Carr

**Affiliations:** ^1^Peninsula Prosthodontics, 19365 7th Avenue NE, Suite 114, Poulsbo, WA 98370, USA; ^2^Physician Assistant Program, University of Charleston, 2300 MacCorkle Avenue SE, Riggleman Hall 130, Charleston, WV 25304, USA; ^3^Department of Health Sciences Research, Mayo Clinic, 200 First Street SW, Rochester, MN 55905, USA; ^4^Department of Pathology, Mayo Clinic, 200 First Street SW, Rochester, MN 55905, USA; ^5^Department of Dental Specialties, Mayo Clinic, 200 First Street SW, Rochester, MN 55905, USA; ^6^Department of Biochemistry and Molecular Biology, Mayo Clinic, 200 First Street SW, Rochester, MN 55905, USA

## Abstract

Oral cancer is often diagnosed only at advanced stages due to a lack of reliable disease markers. The purpose of this study was to determine if the epithelial-specific human calmodulin-like protein (CALML3) could be used as marker for the various phases of oral tumor progression. Immunohistochemical analysis using an affinity-purified CALML3 antibody was performed on biopsy-confirmed oral tissue samples representing these phases. A total of 90 tissue specimens were derived from 52 patients. Each specimen was analyzed in the superficial and basal mucosal cell layers for overall staining and staining of cellular subcompartments. CALML3 was strongly expressed in benign oral mucosal cells with downregulation of expression as squamous cells progress to invasive carcinoma. Based on the Cochran-Armitage test for trend, expression in the nucleus and at the cytoplasmic membrane significantly decreased with increasing disease severity. Chi-square test showed that benign tissue specimens had significantly more expression compared to dysplasia/CIS and invasive specimens. Dysplasia/CIS tissue had significantly more expression than invasive tissue. We conclude that CALML3 is expressed in benign oral mucosal cells with a statistically significant trend in downregulation as tumorigenesis occurs. CALML3 may thus be a sensitive new marker for oral cancer screening.

## 1. Introduction

 Approximately 30,000 new cases of cancer in the oral cavity and oropharynx are diagnosed in the United States each year, corresponding to about 3% of all malignant tumors. Although visibly detectable due to the accessibility of the oral cavity, oral cancer has a high morbidity and mortality because it is typically at an advanced stage when it is finally clinically visible. Accordingly, the five-year survival rate for all stages combined is only 51%. Of the cancers involving the oral cavity and oropharynx, more than 90% are squamous cell carcinomas [1–3]. Up to 40% of those diagnosed with squamous cell carcinoma will develop a metastatic tumor or a second primary tumor to a nearby organ at a later time.

Early diagnosis of oral and oropharyngeal squamous cell carcinoma is crucial for a more favorable prognosis. The oral cavity and oropharynx are easily accessible for routine screening for squamous cell carcinoma, but reliable detection in early stages will require robust markers that are easy to assay. Methods for testing oral epithelial cells for malignancy traditionally involve an invasive and often painful biopsy. Whether as a diagnostic tool or prognostic marker, the potential to develop a simple, less invasive method for oral cancer screening would be of great value.

Human calmodulin-like protein CALML3 (synonyms: CLP, calmodulin-related protein NB-1) is a 148-amino-acid-residue calcium sensor protein closely related to the ubiquitous calmodulin [4, 5]. However, in contrast to calmodulin, CALML3 is tissue specific and seems to be expressed almost exclusively in normally differentiating epithelia such as those of breast, thyroid, prostate, kidney, and skin [6]. In a study comparing normal reduction mammoplasty specimens to archival breast cancer specimens, Rogers et al. [7] found that CALML3 was expressed at high levels in normal breast epithelium and that significant CALML3 downregulation occurred in 79% to 88% of invasive ductal carcinoma and lobular carcinoma specimens. The authors concluded that CALML3 downregulation is common in breast tumorigenesis and that the status of CALML3 expression can serve as a marker for the nonmalignant state [7]. 

We have previously found that CALML3 is expressed at the mRNA transcript level and can be detected by immunohistochemistry in normal oral mucosa tissue. By contrast, there was a notable reduction in immuno staining in areas of malignant transformation [8]. Here we performed immunohistochemical analysis to compare CALML3 expression in tissue samples representing the various phases of oral tumor progression to see if there is a statistically significant trend of changes in CALML3 expression during disease progression. In addition, direct comparisons in CALML3 expression were made between the individual categories of benign, dysplasia, carcinoma in situ, and invasive squamous cell carcinoma. 

## 2. Materials and Methods

### 2.1. Tissue Specimens

Under IRB-approved protocol 754-04, the Mayo Clinic Tissue Registry was searched for patients that had undergone surgical biopsy of the oral cavity. A database was compiled placing the tissue specimens into 4 categories: benign squamous mucosa (including mild reactivity and hyperplasia), squamous dysplasia, squamous cell carcinoma in-situ (CIS), and invasive squamous cell carcinoma. The invasive squamous cell carcinoma was further categorized by grade [9]. The database was cross-referenced for patients who had given research consent. H&E stained slides for the surgical procedures were evaluated and the most appropriate tissue block for each case was processed for CALML3 immunostaining. A staff surgical pathologist (Thomas J. Sebo) confirmed the diagnosis of each sample. Samples were eliminated if there were any questionable diagnoses or if the quality of the section was poor. 

A total of 90 tissue specimens were derived from 52 patients with 62 surgical procedures. For the purpose of this study, the assumption was made that multiple specimens from the same patient are independent. Specimens from the same patient were generally from different lesion sites taken during more than one date of surgery. Among the 52 patients, 31 (60%) were male and the overall mean age at the time of diagnosis was 64 years (SD, 15; median, 67; range, 6 months–87 years). Among the 90 tissue specimens, 30 (33%) were diagnosed as benign, 6 (7%) as dysplastic, 12 (13%) as carcinoma in situ, and 42 (47%) as invasive squamous cell carcinoma. Of the 42 samples diagnosed as squamous cell carcinoma, 2 (5%) were grade 1, 11 (26%) were grade 2, 27 (64%) were grade 3, and 2 (5%) were grade 4. Other data recorded included location of specimen (tongue, floor of mouth, right, left, etc.) and type of surgery (excision versus biopsy).

### 2.2. Immunohistochemistry

 The generation, affinity purification, and characterization of a rabbit polyclonal antibody (TG7) against human CALML3 have been described [7]. These antibodies (generated by Cocalico, Inc., Reamstown) recognize a peptide corresponding to the C-terminal residues 127 to 148 of CALML3 (the most divergent region between CALML3 and calmodulin). Affinity-purified antibodies showed excellent sensitivity and specificity for CALML3 [5, 7]. The paraffin-embedded tissue specimens were cut at a thickness of 6 *μ*m, mounted on positively charged slides, deparaffinized, and treated with H_2_O_2_/methanol to block endogenous peroxidase activity essentially as described [10, 11]. The specimens received heat-induced epitope retrieval in 1 mM EDTA at a pH of 8.0 for 20 minutes. Nonspecific protein-binding sites were blocked in 5% normal goat serum in phosphate-buffered saline solution (PBS)/0.05% Tween-20 for 1 h, and the sections were immunostained by sequential incubations (1-2 h at room temperature each) in affinity-purified CALML3 antibody TG7 (40 *μ*g/mL in PBS/0.05% Tween-20/1% normal goat serum), biotinylated goat anti-rabbit IgG (1 : 200 in PBS/0.05% Tween-20/1% normal goat serum, DAKO, Carpinteria, CA), and horseradish peroxidase-conjugated streptavidin (1 : 300, DAKO, Carpinteria) essentially as described [7]. Slides were rinsed 3 × 5 min in PBS/0.05% Tween-20 between incubations. The sections were then incubated in 3-amino-9-ethylcarbazole in the presence of H_2_O_2_ and counterstained with hematoxylin and mounted with coverslips.

Two observers (a staff surgical pathologist (Thomas J. Sebo) and a maxillofacial prosthodontist (Michael D. Brooks)) evaluated the specimens for expression of CALML3. Staining patterns and intensity were observed and recorded for all categories of samples (benign, dysplasia/carcinoma in-situ, and invasive squamous cell carcinoma). All slides were evaluated at the same magnification. For each specimen, 3–5 separate fields of view were analyzed in 2 mucosal cell layers: superficial cells and basal cells. Analysis of staining in cellular compartments was further subdivided into cytoplasmic membrane (CM), cytoplasm (C), and nucleus (N). Staining was graded on a 4-point scale: 0: absent (no staining above background of a negative control incubated with anti-CALML3 IgG preabsorbed with CALML3 [6]), 1: weak (barely above background and no strong staining in specific cellular regions), 2: intermediate (moderate staining with varying degree of subcellular staining), 3: strong (intense staining with distinct cellular localization). The cytoplasmic membrane, cytoplasm, and nuclei for each mucosal cell region were individually assigned a grade. 

Assigning a grade to each specimen type followed a few basic and logical rules. First, the squamous cells were initially evaluated using two low magnification lenses (2.5x and 5.0x for a total magnification strength of 25x and 50x) to obtain a general sense of staining intensity of all the squamous cells on the slide. Second, for cytoplasmic membrane staining, cells either exhibited circumferential membrane staining or no membrane staining. Third, membrane staining, apart from cytoplasmic staining, was typically most readily apparent in the intermediate layer of the squamous mucosal cells. As the cells reach the actual superficial layer of, for example, benign mucosa and become flattened, the ability to distinguish cytoplasmic staining from cytoplasmic membrane staining was limited. 

Invariably, tissue samples in which no CALML3 staining in the squamous cells was detected could very quickly be assigned a grade of 0 (no staining). In the situation of superficial squamous cell staining, this occurred in 25%–50% of the case material. Conversely, strong (3+) CALML3 immunostaining could be readily ascertained using the same approach in 5%–30% of the case material. In evaluating basally positioned squamous cells, no staining could be detected very quickly in 50% to almost 100% of the case material, whereas strong (3+) staining was very rare (about 2%).

The challenge in this study, and all studies in which immunostain intensity is visually graded, came in grading squamous cells as displaying either weak (1+) or intermediate (2+) CALML3 expression. In these instances, an overall assessment of staining intensity at low magnification showed that the degree of staining could not clearly be categorized as 0 or 3+, and a close analysis of all squamous cells in the tissue sample was performed using all magnification lenses (2.5x, 5x, 10x, 20x, and 40x for overall magnification of 25x, 50x, 100x, 200x, and 400x) to arrive at a grade reflecting the staining intensity for all squamous cells. Thus, a grade of 1+ (weak) indicates that the overall expression of the squamous cells was weak, showing only barely visible staining above 0. For an intermediate grade (2+), the degree of CALML3 staining was felt to be unquestionably present but not as visually strong as a grade of 3+. 

On occasion, rare cells within any given tissue specimen graded as 1+ or 2+ showed strong (3+) or no (0) expression. However, no effort was made to further stratify case material on the basis of the percentage of cells showing a range of CALML3 expressions. As such, our grading system reflects an overall score of CALML3 staining intensity. In those instances in which the two reviewers (Thomas J. Sebo and Michael D. Brooks) recorded different grades for a given case, rereview of the case material was undertaken to arrive at a consensus grade. This occurred in roughly 10% of the case material.

### 2.3. Statistical Methods

 For analysis purposes, grades 0 and 1 were collapsed and reported together as no/weak staining and grades 2 and 3 were collapsed and reported together as intermediate/strong stain. This is because visually weak staining most closely approximates no (0) staining and intermediate (2+) staining most closely approximates strong (3+) staining. Total stain scores were derived by summing the stain grades for the three cellular compartments. The Cochran-Armitage test for trend was used to evaluate whether the proportion of specimens with intermediate/strong expression significantly changed with increasing disease severity. In addition, the chi-square test (or the Fisher's exact test, as appropriate) was used to compare the proportion of specimens with intermediate/strong expression between the benign, dysplasia/CIS, and invasive tissue groups. The Kruskal-Wallis test was used to compare the total stain scores between the three groups. If the *P* value for the overall test of group differences was <0.05, then pair-wise comparisons among the three groups were performed. The analyses assumed that multiple specimens from the same patient were independent. All calculated *P* values were two-sided and *P* values less than 0.05 were considered statistically significant. A Bonferroni correction could be applied by assessing the pair-wise comparisons between the three tissue types using an alpha level of 0.0167 to account for the three comparisons. Statistical analysis was performed using the SAS software package (SAS Institute, Cary, NC). 

## 3. Results

**Figure 1:**
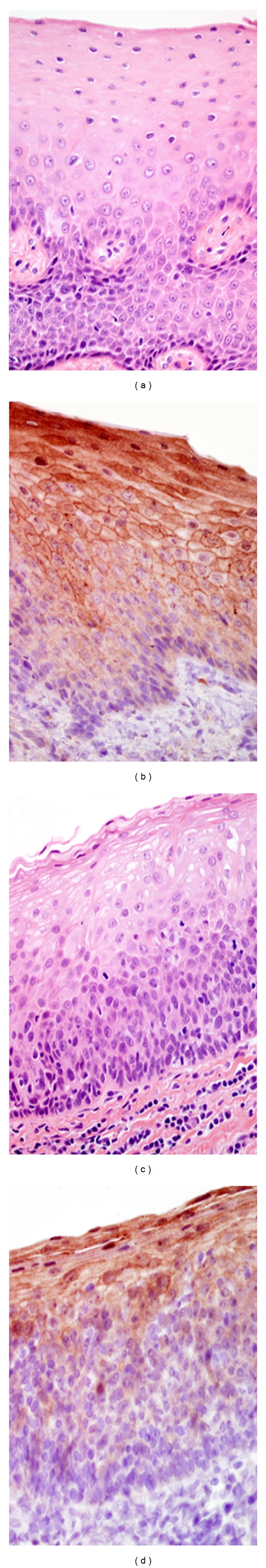
H&E (a) and CALML3 immunostained (b) sections of benign oral epithelium and H&E (c) and CALML3 immunostained (d) sections of dysplastic oral mucosa under high power light microscopy (200x). Immunostaining is more intense towards the superficial layers with an apparent lack of CALML3 expression from the more basaloid cells. Dense cytoplasmic membrane staining and nuclear staining can be appreciated.

**Figure 2:**
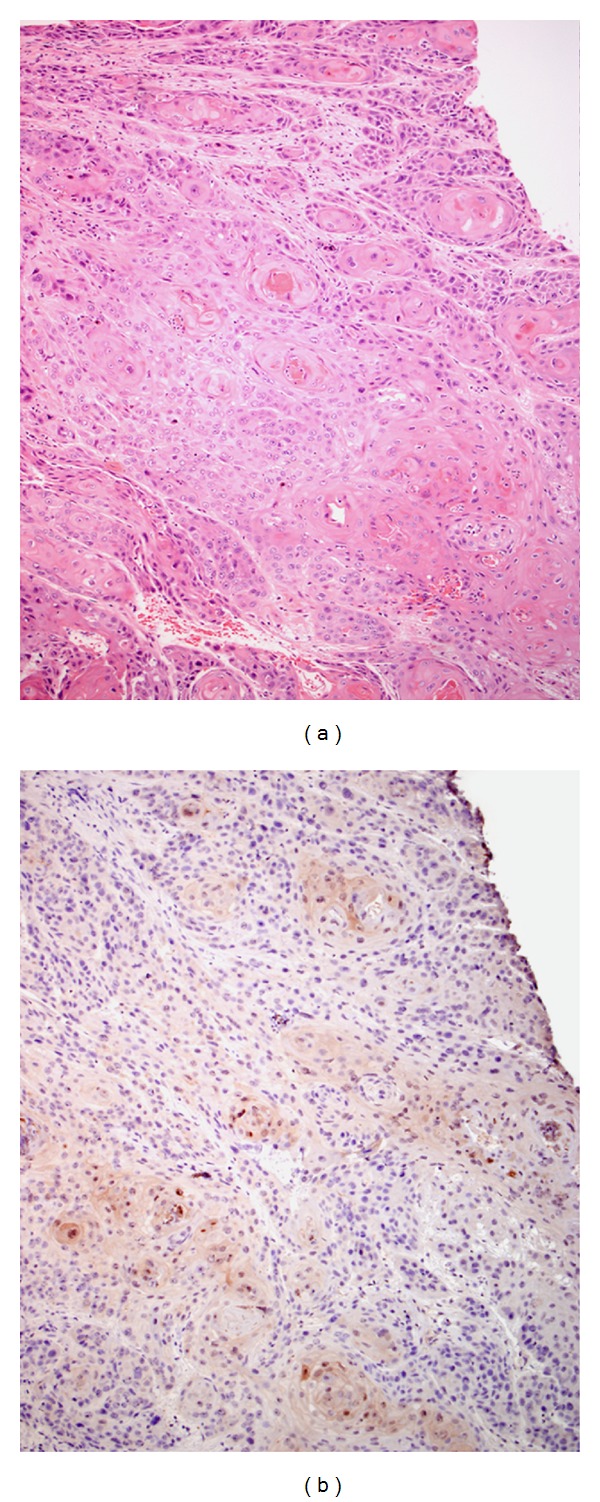
H&E (a) and CALML3 immunostained (b) sections of invasive squamous cell carcinoma under low power light microscopy (100x). An appreciably diminished, and in many areas, absent immunoreactivity is seen in invasive squamous cell carcinoma.

**Figure 3:**
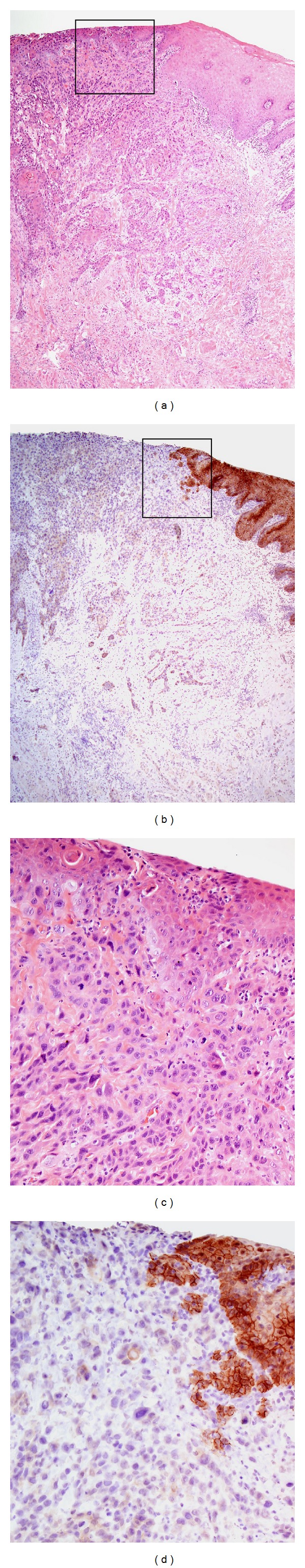
H&E (a) and CALML3 immunostained (b) sections of invasive squamous cell carcinoma with focal area of benign squamous mucosa under low power light microscopy (40x) and H&E (c) and CALML3 immunostained (d) sections of invasive squamous cell carcinoma with focal area of benign squamous mucosa under high power light microscopy (200x). Areas of invasive squamous cell carcinoma show a marked decrease in immunoreactivity ((b) and (d), left side of panels). Areas of intact benign oral mucosa maintain CALML3 expression with notable immunoreactivity (upper right in (c) and (d)).

As we expected from previous studies [6, 8], benign mucosa displayed robust immunostaining (Figures 1(a) and 1(b)). Distinct staining was noted in the periphery (cytoplasmic membrane) and nuclei of the more superficially located epithelial cells (Figure 1(b)). On the other hand, in samples with dysplasia (Figures 1(c) and 1(d)) and carcinoma in-situ (not shown), a marked decrease was seen in strength of stain. A strong reduction (or complete lack) of CALML3 immunostaining was seen in invasive squamous cell carcinoma compared to both benign tissue and dysplasia/CIS tissue (Figures 2 and 3), although in low-grade invasive squamous cell carcinoma, keratin pearls did illustrate mild immunoreactivity. The basal cell layers in general did not stain as intensely as the more superficial cell layers, and no statistical difference was seen with basal nuclear and cytoplasmic staining among the groups. 

Table 1 summarizes the CALML3 expression results at the superficial and basal mucosa levels, separately for the tissue types. Because similar results were found for the dysplastic and CIS specimens, these two tissue types were combined for the analysis. In addition, we found that aside from the superficial cytoplasmic membranes and nuclei of the benign specimens, only a small percentage of the other tissue regions exhibited strong expression. Therefore to evaluate whether there was a trend in the expression level with tissue disease severity, the specimens with weak or no staining were combined, as were those with intermediate or strong staining (see Table 1). However, the detailed summary of all staining results broken down by individual tissue type is available upon request. 

Based on the Cochran-Armitage test for trend, the CALML3 expression level significantly decreased with increasing disease severity for the following sites: superficial cytoplasmic membrane (*P* < 0.001), superficial nucleus (*P* < 0.001), and basal cytoplasmic membrane (*P* = 0.009). On the other hand, a statistically significant trend was not observed for superficial cytoplasm (*P* = 0.19) and basal cytoplasm (*P* = 0.19), in which several dysplastic/CIS specimens had stronger expression than the benign tissue. None of the basal nucleus sites had a stronger than “weak” staining score for any of the tissue types.

**Figure 4:**
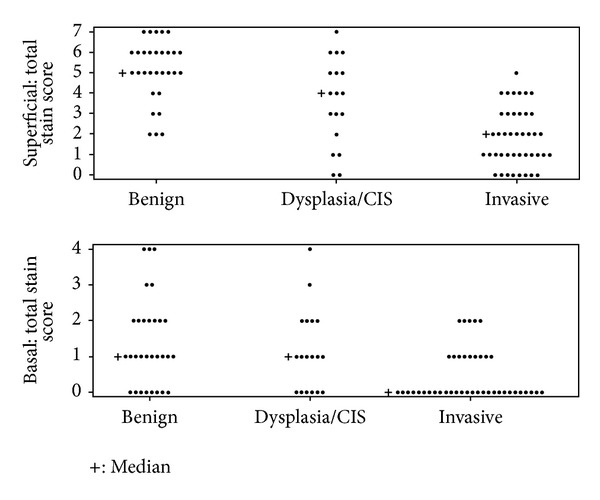
Total stain scores in the superficial cellular compartments and the basal cellular compartments among the tissue categories of benign, dysplasia/carcinoma in situ, and invasive squamous cell carcinoma. A statistically significant trend is seen in CALML3 expression from benign mucosal cells to invasive squamous cell carcinoma. As disease severity increases, CALML3 expression decreases.

The chi-square test (or the Fisher's exact test, where appropriate) was used to compare the proportion of specimens with an intermediate or strong stain between the benign, dysplasia/CIS, and invasive tissue groups (Table 1). Benign tissue specimens had significantly more CALML3 expression in the superficial mucosa compared to invasive specimens (*P* < 0.001) and dysplasia/CIS specimens (*P* = 0.013), as revealed by a comparison of the total stain scores (Table 1 and Figure 4). This difference in CALML3 staining was most pronounced for the cytoplasmic membrane and the nucleus. Dysplasia/CIS tissue also had significantly (*P* = 0.003) more expression than invasive tissue in the superficial mucosa (Table 1 and Figure 4), and this difference was most clearly demonstrated in the cytoplasm (50% versus 7.1%, *P* < 0.001) and in the nuclei (38.9% versus 2.4%, *P* < 0.001). All of the previously outlined comparisons of expression in the superficial layer between the three tissue groups retained statistical significance if a Bonferroni correction was applied and significance is assessed using an alpha level of 0.0167 instead of 0.05.

Expression was not strong in any of the sites in the basal layer (Table 1), though there was evidence suggesting a slight increase in expression in the cytoplasmic membrane of benign squamous cells compared to invasive carcinoma (13.3% versus 0%, *P* = 0.027). In analyzing the 42 specimens of squamous cell carcinoma separately by grade, no obvious trends were apparent. 

## 4. Discussion

Previously, loss of CALML3 immunoreactivity has been linked to early breast cancer development [7]. Here, we show that while CALML3 is strongly expressed in the superficial layers of benign oral mucosa, different oral mucosa tissue types representing the various stages of carcinogenesis exhibit a reduction of CALML3 expression as squamous cells progress from benign, to dysplastic, to carcinoma in situ, to invasive squamous cell carcinoma.

In general, a trend is seen that as disease severity increases, CALML3 expression decreases. The more differentiated the cells, the more intense the staining. Many of the benign specimens showed an increase in staining intensity the further the cells moved away from the basal cell layer. The cells in the basal layer are less mature. As the cells mature, they differentiate and migrate to the upper layers as underlying cells develop. The more intense CALML3 staining towards the outer layers of mucosa is consistent with previous reports that noted markedly increased staining in more differentiated layers of stratified epithelia [6]. Therefore, CALML3 may play a role in terminal differentiation of oral keratinocytes, as has been proposed for the basal and suprabasal keratinocytes of the epidermis during wound healing [12]. CALML3 is a calcium-sensor protein related to calmodulin and as such is thought to exert its function by regulating specific target proteins. Among these, the unconventional myosin-X [13] is known to be involved in directional cell migration and cell adhesion, both are important events during terminal keratinocyte differentiation. The staining for CALML3 in the cytoplasmic membrane of superficial cells in benign oral mucosa may thus reflect its function in regulating MyoX concentrated at the cell periphery [14]. Like calmodulin, CALML3 has multiple targets, one of which (IQ motif containing protein E or IQCE) was recently identified in a yeast two-hybrid screen and may be a protein involved in DNA metabolism (Richard D. Bennett and Emanuel E. Strehler, unpublished). Such a putative nuclear CALML3 target protein(s) could help explain the nuclear staining for CALML3 that we observed in the superficial cells of the normal oral mucosa. However, additional studies are needed to determine the role of CALML3 in the nucleus and its relevance to normal epithelial differentiation. 

Only few diagnostic tests are currently available for oral cancer. Techniques being implemented for diagnostic tests include transepithelial brush biopsy/exfoliative cytology, fine needle aspiration, and toluidine blue [15–23]. These techniques all rely on cytological markers to indicate the presence of malignant or premalignant cells. Although these tests are preferred over conventional biopsy confirmation because they are less invasive and more tolerable for the patient, their reliability and efficacy have not been validated [24–26]. Because the specific expression and cellular localization of CALML3 indicate normal cell function, the potential for development of a simple diagnostic test for oral cancer using CALML3 as a marker is promising. Currently diagnostic screening tools are being utilized to visualize autofluorescence in normal oral tissue with loss of autofluorescence indicative of tissue change or abnormality. Noninvasive screening could be implemented by the use of exfoliative brushing cytology, minibiopsy, or a “swish-and-spit” procedure. This would allow a clinician who observes a suspicious area in the mouth to routinely test for CALML3 levels. A change in CALML3 expression could indicate the presence or the early developing stage of oral cancer.

## Figures and Tables

**Table 1:** Summary of staining results, by tissue type.

Cellular compartment	Tissue type			*P* value^‡^			
	Benign (*N* = 30)	Dysplasia/CIS (*N* = 18)	Invasive (*N* = 42)	Any of the 3 tissue types	Benign versus Dys/CIS	Benign versus invasive	Dys/CIS versus invasive
Superficial: cytoplasmic membrane				<0.001	<0.001	<0.001	0.99
No/weak stain	5 (16.7%)	14 (77.8%)	32 (76.2%)				
Intermediate/strong stain	25 (83.3%)	4 (22.2%)	10 (23.8%)				
Superficial: cytoplasm				<0.001	0.014	0.26	<0.001
No/weak stain	25 (83.3%)	9 (50%)	39 (92.9%)				
Intermediate/strong stain	5 (16.7%)	9 (50%)	3 (7.1%)				
Superficial: nucleus				<0.001	0.23	<0.001	<0.001
No/weak stain	13 (43.3%)	11 (61.1%)	41 (97.6%)				
Intermediate/strong stain	17 (56.7%)	7 (38.9%)	1 (2.4%)				
Superficial: total stain score^†^							
Median (IQR)	5 (5, 6)	4 (2, 5)	2 (1, 3)	<0.001	0.013	<0.001	0.003
Basal: cytoplasmic membrane				0.022	0.28	0.027	—
No/weak stain	26 (86.7%)	18 (100%)	42 (100%)				
Intermediate/strong stain	4 (13.3%)	0 (0%)	0 (0%)				
Basal: cytoplasm				0.13	—	—	—
No/weak stain	27 (90%)	15 (83.3%)	41 (97.6%)				
Intermediate/strong stain	3 (10%)	3 (16.7%)	1 (2.4%)				
Basal: nucleus				0.99	—	—	—
No/weak stain	30 (100%)	18 (100%)	42 (100%)				
Intermediate/strong stain	0	0	0				
Basal: total stain score^†^							
Median (IQR)	1 (0, 2)	1 (0, 2)	0 (0, 1)	<0.001	0.55	<0.001	0.010

IQR: interquartile range. Values are reported as *N* (%) unless otherwise noted.

^†^Total stain score was derived as the sum of the staining grades for the three components: cytoplasmic membrane, cytoplasm, and nucleus components. Each component was graded on a scale of 0 = absent, 1 = weak, 2 = intermediate, and 3 = strong.

^‡^Comparisons between the three tissues types were evaluated based on the chi-square test or Fisher's exact test for the categorical variables and the Kruskal-Wallis test for the total stain scores. If the *P* value for the overall test for differences was <0.05, then pair-wise comparisons among the three tissue types were performed.
